# A Study of the Effect of the Front-End Styling of Sport Utility Vehicles on Pedestrian Head Injuries

**DOI:** 10.1155/2018/8502507

**Published:** 2018-03-20

**Authors:** Guanjun Zhang, Qin Qin, Zheng Chen, Zhonghao Bai, Libo Cao

**Affiliations:** State Key Laboratory of Advanced Design and Manufacturing for Vehicle Body, Hunan University, 1st Lushan South Street, Changsha 410082, China

## Abstract

**Background:**

The number of sport utility vehicles (SUVs) on China market is continuously increasing. It is necessary to investigate the relationships between the front-end styling features of SUVs and head injuries at the styling design stage for improving the pedestrian protection performance and product development efficiency.

**Methods:**

Styling feature parameters were extracted from the SUV side contour line. And simplified finite element models were established based on the 78 SUV side contour lines. Pedestrian headform impact simulations were performed and validated. The head injury criterion of 15 ms (HIC_15_) at four wrap-around distances was obtained. A multiple linear regression analysis method was employed to describe the relationships between the styling feature parameters and the HIC_15_ at each impact point.

**Results:**

The relationship between the selected styling features and the HIC_15_ showed reasonable correlations, and the regression models and the selected independent variables showed statistical significance.

**Conclusions:**

The regression equations obtained by multiple linear regression can be used to assess the performance of SUV styling in protecting pedestrians' heads and provide styling designers with technical guidance regarding their artistic creations.

## 1. Introduction

As vulnerable road users, pedestrians have a high risk of severe injury or fatality in traffic accidents. According to statistical data from the World Health Organization [1], pedestrian fatalities accounted for 22% of the total fatalities caused by road traffic accidents worldwide in 2013. Due to the rapid increase in the number of motor vehicles and the mixed pedestrian-vehicle road traffic environment, China faces an even more severe pedestrian safety problem. In 2012, pedestrian fatalities accounted for approximately 25% of the total fatalities caused by traffic accidents in China [2]. In a vehicle-to-pedestrian crash, the head of the pedestrian is the body part that is most susceptible to fatal injuries [3]. A rapid increase in the market shares of sport utility vehicles (SUVs) has occurred in recent years throughout the world and in China [4, 5]. Compared with cars, SUVs may cause a larger number of fatal injuries to pedestrians due to their high front-end structures and relatively high collision energy [6]. Therefore, the effect of SUVs on pedestrian head injuries needs to be investigated to substantially improve the pedestrian collision safety performance of these vehicles.

To improve vehicle performance in protecting pedestrian heads during crashes, numerous researchers have conducted extensive studies of the structure design and styling of vehicles. Some studies reported that pedestrian protection performance can be improved by modifying the stiffness of the hood inner plate or adding a collapsible hinge [7, 8]. Several researchers also employed the design optimization approach of hood panels to reduce pedestrian head injuries [9, 10]. However, these vehicle-body-structure-based improvement measures can only be implemented after the styling design stage. Some researchers believed that the headform test zones should avoid certain danger points (e.g., the junctions of the hood and the headlights, the junctions of the hood and the fenders, the areas where the hood hinges and latches were installed, the wiper shafts, and the ventilation cover) to improve the headform impact test score during the initial styling design stage, according to the requirements of the division of headform test zones in pedestrian protection regulations [7, 11]. However, these improvement methods were evasion strategies regarding the relevant regulations; they did not analyze the relationship between the front-end styling of vehicles and pedestrian injuries.

Several studies discovered that pedestrian kinematic responses and injury risk were closely related to the front-end geometry of vehicles via the postmortem human surrogate (PMHS) impact test and the total human model for safety (THUMS) impact test, respectively [12, 13]. Yang established vehicle-to-pedestrian impact simulations based on accident reconstructions and concluded that the main parameters of front shape that affected head impact conditions included the bumper lead (BL), bumper center height (BCH), hood leading edge height (HLEH), hood length (HL), and hood angle (HA) [14]. Zhang et al. concretized the parameters of the front-end structures of vehicles and analyzed them based on the Pedestrian Crash Data Study (PCDS); they noted that the wrap-around distance (WAD) of the hood rear edge (WAD_HRE_) had a significant impact on the risk of head injury, with a score of 2+ on the Abbreviated Injury Scale (AIS2+) [15]. Liu et al. investigated the relationship between HA and HIC of headform via finite element (FE) analysis [16]. By analyzing the specific vehicle front structure parameters, the previously mentioned studies have demonstrated that the vehicle front structure parameters have a significant effect on pedestrian injuries. However, few studies have investigated the relationships between the front-end styling features of vehicles and pedestrian head injuries at the styling design stage, which precedes the structure design stage.

Therefore, to consider the pedestrian safety performance of SUVs during the initial styling design stage for reducing pedestrian head injuries, this study characterized SUVs' styling in terms of their styling feature lines, extracted the relevant styling-feature-line-based styling feature parameters, and established simplified FE models for the front-end styling of SUVs based on their styling feature lines. This study also simulated collisions between headform impactors and the front-end styling of SUVs according to the pedestrian testing protocol of the European New Car Assessment Programme (Euro NCAP) and investigated the relationships between the styling feature parameters and pedestrian head injuries using a multiple linear regression analysis method.

## 2. Methods

### 2.1. Sample Selection and Styling Feature Lines

**Figure 1:**
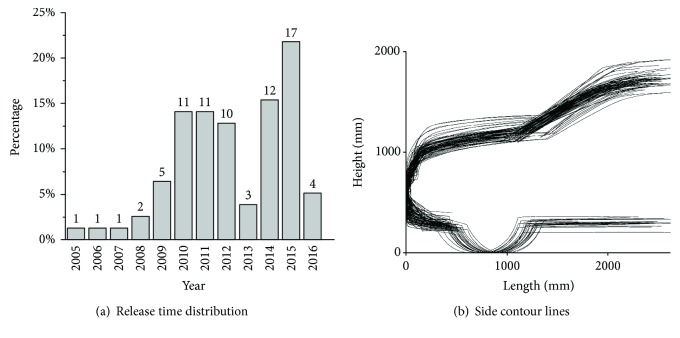
Release time distribution and side contour lines of the SUVs studied in this study.

According to the sales ranking in the Chinese market, 78 SUVs with Euro NCAP test results were selected for this study. These SUVs were released from 2005 to 2016, and the release time distribution was shown in Figure 1(a). The overall length, overall width, and overall height of these SUVs were 4623 ± 212 mm, 1865 ± 66 mm, and 1706 ± 62 mm, respectively (Table 1).

The side contour line (main styling feature line) of a vehicle, which can be extracted from a side-view drawing, is the most important styling feature line of the vehicle. It determines the total feel and style of the vehicle and is the main feature line determined during the styling design stage [17]. Therefore, this study primarily investigated the relationship between the main styling feature line and pedestrian head injuries. Because the front structures (bumper, grille, and hood) of a vehicle compose the main parts that come in contact with a pedestrian during a collision, only the front section of the side contour line of each sampled SUV model was investigated in this study. The side contour line of a SUV was extracted from its side view and then was scaled to its actual size based on overall length and height in AutoCAD software (AutoCAD 2010, Autodesk Inc., San Rafael, CA). Seventy-eight side contour lines were illustrated in Figure 1(b).

### 2.2. Extraction of Styling Feature Parameters

**Figure 2:**
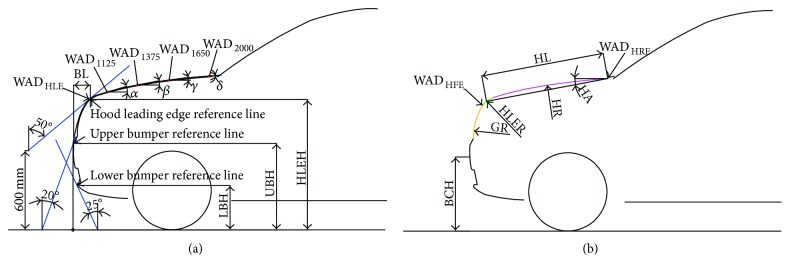
Front-end styling feature parameters of the SUV models. (a) Styling feature parameters based on the Euro NCAP protocol. (b) Styling feature parameters based on the vehicle structure.

Nine styling feature parameters were defined according to the Euro NCAP's pedestrian testing protocol (Figure 2(a)) [18]. Eight real structure parameters were also defined based on the actual structures of the vehicles (Figure 2(b)). Thus, a total of 17 styling feature parameters were extracted (refer to Table 2 for detailed descriptions). All styling feature parameters were measured with AutoCAD software.

### 2.3. Establishment and Verification of Simplified FE Models of the Selected SUVs

**Figure 3:**
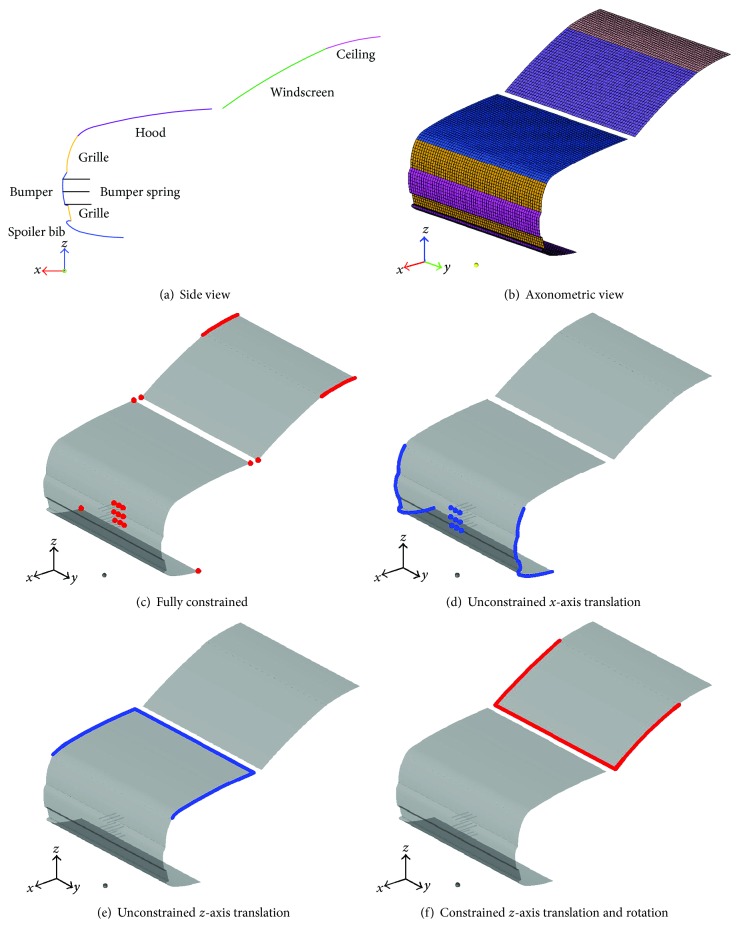
Simplified FE model of a typical SUV model and the relevant constraint settings.

According to actual vehicle structures, components of the spoiler, bumper, grille, hood, windshield, and ceiling for each extracted styling feature line were established (Figure 3). To study the effect of the styling features on pedestrian head injuries, the boundary conditions for all SUV FE models were set to be the same, and the same components of the models were assigned with the same material parameters, element properties, and element thickness. Each FE model also has the same number of nodes and elements and mesh distribution. And the vehicle widths were uniformly set to 1790 mm for the SUV models. The element size of all model was about 30 mm. To accurately reflect the kinematic responses of the legform impactor, nine spring elements (3 × 3) were placed behind the impactor-bumper collision area, where they were evenly distributed throughout the middle section of the bumper to absorb energy. On the *y*-axis, adjacent spring elements were spaced at a distance of 60 mm. On the *z*-axis, the spring elements were evenly distributed across the width of the bumper. The spring stiffness was determined based on a previous study [19], and the same parameters were set for the spring elements in all FE models.

Suitable constraints were exerted on the left and right edges of each simplified FE model to better simulate the collision responses of all impactors. The constraints were applied as follows. (1) To position and support each simplified FE model, full constraints were exerted on the corner points and the rear-end points of the bumper spring elements (Figure 3(c)). (2) To simulate the motion tendency of the bumper, spoiler, and grille, as well as the front-end points of the spring elements in the *x* direction during a collision between the lower leg impactor and an SUV, with the exception of the translation in the *x* direction, all degrees of freedom of the boundary nodes of the bumper, spoiler, and grille, as well as the front-end points of the spring elements, were constrained (Figure 3(d)). (3) To simulate the motion tendency of the hood in the *z* direction, only the *z* direction translation degree of freedom of the edge nodes of the hood was allowed (Figure 3(e)). (4) To simulate the rigid boundaries and motion tendency of the windshield, the translation and rotation degrees of freedom of the edge nodes of the windshield in the *z* direction were constrained (Figure 3(f)).

In accordance with the Euro NCAP's pedestrian testing protocol and assessment protocol (version 5.3.1) [18], four headform-to-vehicle collisions were simulated for each of the SUV models. The points of WAD_1125_, WAD_1375_, and WAD_1650_ were selected as the child headform impact points, and the WAD_2000_ point was selected as the adult headform impact point. If the WAD_2000_ point is located in one of the dangerous areas for collision (e.g., the wipers and the lower edge of the windshield), the HIC_15_ value at WAD_2000_ is considered to exceed the limit [18]. For the adult headform impact test, therefore, only the SUV models where the WAD_2000_ points were located on the hood were considered in this study (48 SUV models satisfied this condition). An Arup 3.5 kg child pedestrian headform model was employed as the child headform impactor. An Arup 4.5 kg adult pedestrian headform model was employed as the adult headform impactor. The pedestrian test results of 20 SUV models that were published on the official website of the Euro NCAP were used to verify the FE models. Because the extracted side contour line of each SUV model was taken from the longitudinal central axis of the vehicle, only the Euro NCAP's test results for the eight impact areas (four left impact areas and four right impact areas) on the hood immediately adjacent to the longitudinal central axis of the vehicle were used to verify the simplified FE models. The explicit code from the LS-DYNA software package (LSTC, Livermore, CA) was employed as the FE solver in this study.

### 2.4. Statistical Method

With the styling feature parameters treated as the independent variables (a total of 17 independent variables) and the HIC_15_ values in the four impact points obtained from the collision simulations treated as the dependent variables, a multiple linear regression analysis method was employed to describe the relationships between the styling feature parameters and the HIC_15_ value at each impact point. A stepwise regression method was employed for independent variable selection. Because multiple linear regression requires that the linearity, independency, normal distribution, and equal variance conditions (i.e., the LINE conditions) must be satisfied, this study examined whether the samples satisfy the LINE conditions based on residuals analysis. Multicollinearity was diagnosed using a variance inflation factor (VIF) < 4 or a tolerance > 0.25 to ensure that no collinearity occurred between the independent variables, that is, that the independent variables were independent. In addition, outlier detection was conducted, and outlier samples with a residual error > 2 were eliminated in the studentized residual error plot to prevent a large value of dispersion of the sample data, which would produce a relatively large residual error and consequently affect the goodness of fit (*R*^2^) of the regression equation. All statistical analysis procedures were performed using the statistical software SPSS (IBM Corporation, Somers, NY).

## 3. Results

### 3.1. Model Validation Results

The validation results of simplified FE models were explained using the 2011 BMW X3 model as an example.  Table 3 lists the Euro NCAP's test results for the four left test areas and the four right test areas (adjacent to the longitudinal central axis) and the simulation results for this SUV model. The simulation results of the WAD_1000_-WAD_1250_, WAD_1250_-WAD_1500_, and WAD_1500_-WAD_1800_ impact areas were consistent (the same color as) with the Euro NCAP's test results for both sides. The HIC_15_ from simulation for the WAD_1800_-WAD_2100_ impact area was 900 (green), which was consistent with the Euro NCAP's test result on the right side of the vehicle (green), but inconsistent with the result on the left side of the car (yellow). This may be related to the specific components under the hood, because the current study focused on the styling without considering the specific structure, and the main styling feature line was in the longitudinal symmetry of the car. It can be considered that the 2011 BMW X3 simplified FE model could predict the impact response of the headform to the hood.

Twenty tested SUV models were employed for the validation. When the simulation results were consistent (the same color as) with the Euro NCAP's test results for at least one side for an impact area, the simulation results were considered to be consistent with the Euro NCAP's test results for this impact area.  Table 4 lists the number of SUV models whose simulation results were consistent (the same color as) with the corresponding Euro NCAP test results in each of the four impact areas and the corresponding simulation accuracy. The simulation results of all impact areas had a minimum accuracy of 90%, with the exception of the simulation results for the child headform impact test in the WAD_1000_-WAD_1250_ impact area, which had an accuracy of only 60%. Of the 20 SUV models, the simulation results were consistent with the Euro NCAP's test results in all four impact areas for ten SUV models. The simulation results were inconsistent with the Euro NCAP's test results in one of the impact areas for seven SUV models, and the simulation results were inconsistent with the Euro NCAP's test results in two of the impact areas for three SUV models. These findings indicated that the simplified FE models adequately reflect the actual collision situations.

### 3.2. Simulation Results

**Figure 4:**
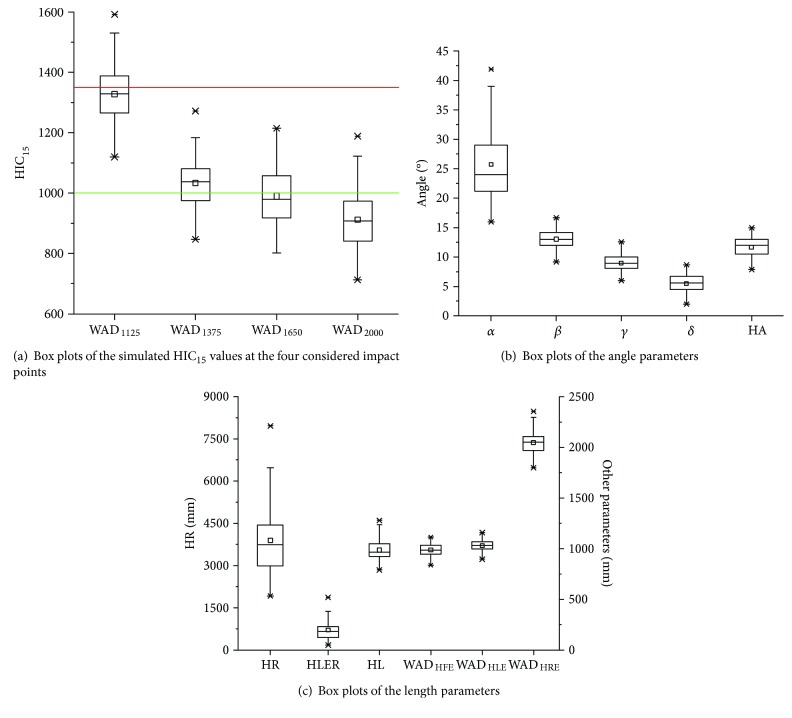
Box plots of the simulated HIC_15_ at the four impact points and the styling feature parameters.

Figure 4(a) shows the box plots of the simulated HIC_15_ values in different impact areas for 78 SUV models. According to the Euro NCAP's pedestrian assessment protocol (version 5.3.1), an HIC_15_ value of ≤1000 corresponds to a full mark, an HIC_15_ value of ≥1350 corresponds to a zero mark, and a value of 1000 < HIC_15_ < 1350 corresponds to a mark between zero and a full mark. Based on the box plots shown in Figure 4(a), the closer the impact point was to the front end of the hood, the higher the HIC_15_ because the front section of the hood has a relatively high stiffness due to structural features such as the angle of the front hood and hood latches. The values of the HIC_15_ at WAD_1375_ (HIC_WAD1375_) and HIC_WAD1650_ ranged from 1000 to 1350, whereas HIC_WAD2000_ primarily remained below 1000. The results indicated that the HIC_15_ decreased with an increase in WAD on the hood.

### 3.3. Multiple Linear Regression Analysis Results

Of the 17 styling feature parameters that were considered in this study, six parameters that were not statistically significant for the four HIC_15_ values should be removed based on the stepwise regression method for multiple linear regression. Thus, a total of 11 styling feature parameters that had a statistical significance for the HIC_15_ values were selected. The box plots of these styling feature parameters are shown in Figures 4(b) and 4(c). When the WAD was short, the angle between the tangent of the hood at the impact point and the horizontal was large. An overlap was observed between the ranges of WAD_HFE_ (853–1100 mm) and WAD_HLE_ (896–1162 mm).

Table 5 lists the *R*^2^ values of the multiple linear regression models for the HIC_15_ values obtained from the headform impact simulations at the four impact points and the results of relevant statistical tests. For example, the regression model for HIC_WAD1125_ had an *R*^2^ of 56.0%. According to the analysis of variance and the *t* test results, the regression model and the selected independent variable coefficients were statistically significant (*F* = 23.199, *p* < 0.001, sig. < 0.05). According to the collinearity test, no collinearity was observed among the independent variables (VIF < 4). According to the standardized coefficients, *α*, WAD_HFE_, HL, and HA were the main factors that affected HIC_WAD1125_; *α* was the primary factor, followed by WAD_HFE_, HL, and HA.

## 4. Discussion

In the design of a vehicle, pedestrian collision safety performance is generally considered during the structure design stage, or modifications are made to the styling or structure of the vehicle to satisfy pedestrian collision regulations. The specific effect of the styling of the vehicle on pedestrian injuries are not considered during the styling design stage. However, considering the effect of vehicle styling on pedestrian injuries during the styling design stage can help to improve both the pedestrian protection performance and the research and development efficiency.

Based on the key styling feature line of a vehicle (the side contour line), the Euro NCAP's pedestrian testing protocol (version 5.3.1), and real vehicle structures, this study defined 17 styling feature parameters that characterize the front-end styling of a SUV. Based on the side contour line, simplified FE models were established for the front-end styling of each of the 78 SUV models. Shell elements were used to simulate the outer surface styling features of each selected SUV model. The approximate characteristics of pedestrian collision responses were realized by imposing constraints on the FE model. The simplified styling-feature-line-based FE model of the front-end styling of each selected SUV model accurately reflected its styling. However, the stiffness of the front-end structure affects the acceleration of the head directly in the vehicle-to-pedestrian collision. The higher the stiffness of the structure, the higher the HIC, indicating a higher head injury risks. The geometry of vehicle outer sheet metal parts and the components under it (such as the engine and hood lock) affect the stiffness of the outer sheet metal parts when it is deformed. Because the FE models were established without considering the specific structures of SUVs, some differences between the results obtained from the simplified FE models and the results obtained in tests conducted on actual SUVs were observed. However, no specific structure has been designed during the styling design stage, and styling designers should not limit themselves by focusing on the body structure of a vehicle when designing its styling. Therefore, the components under the outer cover were not considered in this study, and the boundary conditions for all SUV simplified models were unified so that only the styling factor influenced the head impactor response. The model validation results (Table 4) demonstrated that the simulation results were consistent with the Euro NCAP's test results for half of the 20 SUV models that were considered for validation. In the WAD_1250_-WAD_1500_, WAD_1500_-WAD_1800_, and WAD_1800_-WAD_2100_ impact areas, the simulation results were inconsistent with the Euro NCAP's test results for a maximum of two SUV models. Although the collision responses of the simplified FE models in the WAD_1000_-WAD_1250_ impact area were relatively unsatisfactory, the simulation results of 60% of the SUV models were consistent with the Euro NCAP's test results. Therefore, the simplified FE model of the front-end styling of each selected SUV model based on the styling feature line can not only characterize the front-end styling of an SUV but also simulate the pedestrian headform impact responses, which comprehensively reflects the styling and pedestrian collision safety performance of an SUV. This validation of the FE models based on real vehicle collision test data also indicates that the established simplified FE models reflect real pedestrian headform impact response characteristics. Although they were established based on the styling feature lines, the simplified FE models do not markedly deviate from the actual vehicles with structural characteristics. In addition, the same mesh distribution, material parameters, element properties, constraints, and vehicle width were employed in all simplified FE models to prevent these factors from affecting the simulation results of different SUV models and to enable the statistical analysis results to reflect the effect of the considered styling feature parameters on pedestrian head injuries.

Table 6 lists the positive/negative correlations among the HIC_15_ values at the four considered impact points (WAD_1125_, WAD_1375_, WAD_1650_, and WAD_2000_) and the 11 relevant styling feature parameters and their effect degree (based on the regression results).

**Figure 5:**
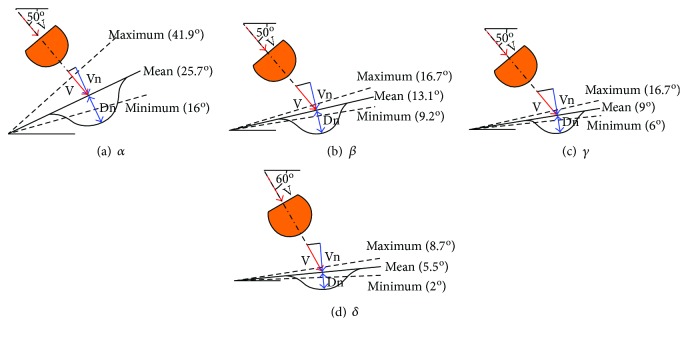
Statistical data on the angles between the tangent of the hood at the four impact points and the horizontal and schematics of the required deformation distance of the hood (produced based on Lawrence et al. [20]).

*α*, *β*, *γ*, and *δ* had a significant effect on and were positively correlated with the HIC_15_ at their respective impact points. The HIC_15_ value increased with an increase in the angle between the tangent of the hood at the impact point and the horizontal. As shown in Figure 5, when the angle between the tangent of the hood at the impact point and the horizontal was small, the velocity component of the headform impactor in the direction normal to the impact point on the hood was small. Thus, a small hood deformation distance is required. For a given impact velocity and angle of the headform impactor, a smaller angle between the tangent of the hood at the impact point and the horizontal can help to reduce the HIC_15_ value. Tables 5 and 6 indicate that *α* was the primary factor that affected HIC_WAD1125_ and that *γ* and *δ* were secondary factors that affected HIC_WAD1650_ and HIC_WAD2000_, respectively. *α* was the largest angle among the four angles (as shown in Figures 4 and 5); thus, the impact angle of the headform impactor at the WAD_1125_ point was closer to 90° relative to the hood. Thus, a larger deformation space and stricter structure design requirements are needed, and the potential for a severe head injury is significant. Consequently, *α* has a significant effect on pedestrian head injuries. This study notes that the angle between the tangent of the side contour line of the hood at every point and the horizontal is a very important factor that affects pedestrian head injuries. Therefore, a slightly rising hood design will help to reduce pedestrian head injuries in the event of a collision.

Yang reported that HLEH, BL, and BCH were the main parameters that affect pedestrian head injuries. This study shows that WAD_HLE_ and WAD_HFE_ have a significant effect on pedestrian head injuries [14]. Peng et al. noted that the WAD on the engine hood was significantly affected by the front-end structure of the vehicle [21]. The results of the Pearson's product-moment correlation coefficient indicated that the correlation coefficients between WAD_HLE_ and WAD_HFE_ and HLEH, BL, and BCH are significant. The correlation coefficient between WAD_HLE_ and HLEH was the highest, with a value of 0.94, and the remaining correlation coefficients were greater than 0.5. In addition, all correlations were positive (Table 7). Therefore, the analysis indicates that the results of the effect of WAD_HLE_ and WAD_HFE_ on the pedestrian head injuries obtained in this study are consistent with the findings of Yang.

**Figure 6:**
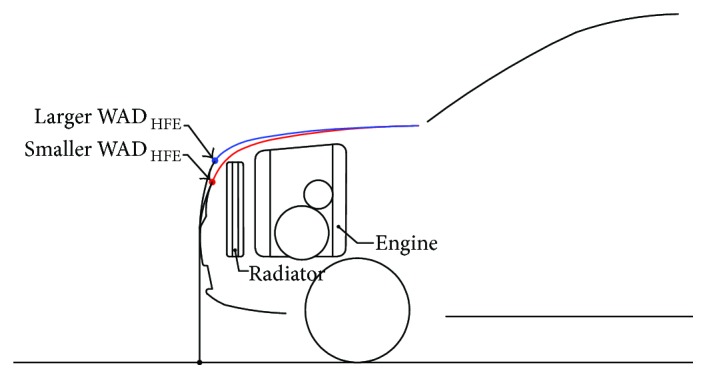
Schematic of a relatively long WAD_HFE_ and a relatively short WAD_HFE_.

The main WADs in the side contour line of the hood (WAD_HLE_, WAD_HFE_, and WAD_HRE_) are important parameters that affect pedestrian head injuries. WAD_HFE_ has the second most significant effect on HIC_WAD1125_ after *α*, and a longer WAD_HFE_ can help to reduce pedestrian head injuries. A long WAD_HFE_ indicates that the front end of the hood is already rather high, which indicates that ensuring sufficient space in the engine compartment into which the hood can deform is easy. In addition, a long WAD_HFE_ indicates that the front end of the hood can extend gradually toward the back of the vehicle. In this case, the front part of the hood can be designed to have a low stiffness, and the hood can also be designed to have a small HA. These design measures are favorable for reducing the HIC_15_ value. Conversely, a short WAD_HFE_ indicates that the front end of the hood is low. In this case, to satisfy the requirements for arranging the necessary components within the engine compartment, the front section of the hood needs to ascend steeply to provide sufficient space in the engine compartment. Consequently, the front section of the hood will have a high stiffness, and ensuring sufficient space for the hood to undergo inward deformation will be difficult. The hood may also have a relatively large HA. These factors are detrimental to the reduction of the HIC_15_ value (Figure 6), which may be the cause of the negative correlation between WAD_HFE_ and HIC_WAD1125_. To improve the performance of SUVs in protecting child pedestrians' heads, WAD_HFE_ should be increased, that is, an overly low front-end design for the hood is inadvisable. In addition, a smaller HA will facilitate better safety performance.

In this study, WAD_HLE_ is the primary factor that affects HIC_WAD1375_ and is positively correlated with HIC_WAD1375_. A longer WAD_HLE_ (896–1162 mm) indicates that the WAD_1375_ impact point is closer to the (leading) edge of the hood. Because the hood has a higher stiffness at its front section, a long WAD_HLE_ can easily produce a high HIC_15_ value. Conversely, WAD_HLE_ has a negative but insignificant correlation with HIC_WAD1650_. A long WAD_HLE_ (896–1162 mm) indicates that the WAD_1650_ impact point is closer to the middle of the hood. Because the hood has a low stiffness in its middle section, a long WAD_HLE_ can yield a small HIC_15_ value. Therefore, WAD_HLE_ should be decreased to improve the total performance of the front and middle sections of the hood of an SUV in protecting pedestrians' heads.

WAD_HFE_ and WAD_HLE_ represent the WADs at the front end of the hood and the leading edge of the hood, respectively. WAD_HFE_ is negatively correlated with the HIC_WAD1125_, and WAD_HLE_ is positively correlated with the HIC_WAD1375_ (Table 6). Although the ranges of WAD_HFE_ and WAD_HLE_ overlap in their box plots (Figure 4(c)), for the majority of a single SUV, the condition of WAD_HFE_ < WAD_HLE_ is satisfied. To reduce pedestrian head injuries, simultaneously increasing WAD_HFE_ and decreasing WAD_HLE_ are not conflicting. In several cases, WAD_HFE_ is longer than WAD_HLE_, which indicates that the hood leading edge may be located on the grille in front of the hood. This situation may be more favorable for improving the performance of an SUV in protecting pedestrians' heads.

WAD_HRE_ (1802–2355 mm) is the primary parameter that affects HIC_WAD2000_ and is also positively correlated with HIC_WAD2000_. Han et al. discovered that longer engine hoods will cause more severe head injuries [13], which is consistent with the finding of this study: a long WAD_HRE_ causes an increase in severe head injuries. The side contour line of each SUV model that was investigated in this study is extracted from the bilateral symmetry plane. The rear edge of the hood on the bilateral symmetry plane lacks any rigid structures, such as support hinges and wipers. In addition, this study only considers situations in which the headform impact point is located on the hood and does not consider situations in which the headform impact point is located on the rear edge of the hood or the windshield. According to the results of this study, reducing WAD_HRE_ when designing the styling of an SUV will help to reduce the HIC_15_ value.

The HA and HL have a significant effect on and are positively correlated with child HIC_15_ values (HIC_WAD1125_ and HIC_WAD1375_). Thus, a larger HA or a longer HL will increase the severity of child head injuries, which is consistent with the findings of the reference [13, 16], respectively. During the development of an SUV model, the HA and HL should be reduced. The HR is the primary factor that affects HIC_WAD1650_ and a tertiary factor that affects HIC_WAD2000_; it is also positively correlated with both HIC_WAD1650_ and HIC_WAD2000_. This finding indicates that when the impact point is closer to the middle section of the hood, the effect of the HR on pedestrian head injury will be more significant because a small HR corresponds to a more pronounced protrusion of the hood and, consequently, a greater gap between the engine and the hood, which can help to reduce the HIC_15_ value. In the styling design of an SUV, reducing the HR can improve its performance in protecting pedestrians' heads.

This study has some limitations. First, the study is based on the main styling feature line of a vehicle, namely, the side contour line (located on the longitudinal symmetry plane of a vehicle), and focuses on the head injury of the pedestrian headform impactor when it impacts the hood of an SUV model on the longitudinal symmetry plane. The performance of other areas of the SUV (e.g., the front windshield), as determined by other styling features, in protecting pedestrians' heads and the effect of the relevant styling features on the impact responses to an upper-leg impactor require additional investigation. A styling design that shows excellent performance in protecting pedestrians' heads may not necessarily perform well in protecting other body parts of pedestrians. Second, this study focuses on the relationships between the styling features of an SUV and its performance in protecting pedestrians, as evaluated using pedestrian impactors. Although the results of this study can help to improve impactor test results for SUVs, additional research involving the use of human FE models is required to determine whether the measures recommended based on this study can also effectively improve the actual pedestrian protection performance of an SUV. Therefore, a mesh morphing method based on radial basis functions will be used to rapidly morph a baseline vehicle frontal structure model into other vehicle frontal geometry targets, so as to quantitatively evaluate the impact of styling features on pedestrian injuries. Last, this study focuses on pedestrian head injuries based on the styling features of a vehicle and does not consider the specific structural design or the materials of the vehicle components. A reasonable structural design and component material optimization can help to improve the pedestrian protection performance of an SUV. Attention should also be paid to the structure feasibility of the styling design.

## 5. Conclusions

This study investigated the relationships between the styling feature parameters based on the side contour lines of SUVs and the HIC_15_ values obtained in pedestrian headform impact tests using FE simulations and stepwise regression analysis. Based on the results, relationships were established between the HIC_15_ values at four impact points and the selected styling feature parameters. Styling feature parameters, such as the angle between the tangent of the hood at the impact point and the horizontal; WAD_HLE_, WAD_HFE_, and WAD_HRE_; and HA, HL, and HR, had a significant effect on pedestrian head injuries. During the styling design of an SUV, reducing the angle between the tangent of the hood at the head impact point on the side contour line and the horizontal, increasing WAD_HFE_, reducing WAD_HLE_ and WAD_HRE_, and reducing HA, HL, and HR can improve an SUV's performance in protecting pedestrians' heads. The regression equations obtained in this study can be used to assess the performance of SUV styling designs in protecting pedestrians' heads during the styling design stage and provide styling designers with technical guidance for their artistic creations.

## Figures and Tables

**Table 1:** Basic parameters of the selected SUV models.

Basic parameters	Statistical results		
	Mean	SD	Range
Length/mm	4623	212	4160–5118
Width/mm	1865	66	1730–2034
Height/mm	1706	62	1583–1882
Wheel base/mm	2728	113	2560–3020
Curb weight/kg	1723	323	1210–2744

**Table 2:** Front-end styling feature parameters of the SUV models and their descriptions.

	Parameter	Description
Parameters based on the Euro NCAP protocol	HLEH	Hood leading edge height
	UBH	Upper bumper height
	LBH	Lower bumper height
	BL	Bumper lead
	WAD_HLE_	Wrap-around distance at the hood leading edge
	*α*	Angle between the tangent of the hood at the WAD_1125_ point and the horizontal
	*β*	Angle between the tangent of the hood at the WAD_1375_ point and the horizontal
	*γ*	Angle between the tangent of the hood at the WAD_1650_ point and the horizontal
	*δ*	Angle between the tangent of the hood at the WAD_2000_ point and the horizontal

Parameters based on vehicle structure	BCH	Bumper center height
	GR	Grille radius
	HLER	Hood leading edge radius
	HA	Hood angle
	HR	Hood radius
	HL	Hood length
	WAD_HFE_	Wrap around distance at the hood front edge
	WAD_HRE_	Wrap around distance at the hood rear edge

**Table 3:** Comparison of the headform impact test results and the simulation results for the 2011 BMW X3 model.

Impact area	Test results (car left)	Test results (car right)	Simulation results (HIC_15_)
WAD_1800_-WAD_2100_	Yellow	Green	Green (900)
WAD_1500_-WAD_1800_	Green	Green	Green (1000)
WAD_1250_-WAD_1500_	Green	Green	Green (959)
WAD_1000_-WAD_1250_	Yellow	Yellow	Yellow (1308)

**Table 4:** Comparison between the simulation results and the test results for 20 SUV models.

Impact area	Injury parameter	Number of models with consistent results	Accuracy
WAD_1800_-WAD_2100_	HIC_15_	18	90%
WAD_1500_-WAD_1800_		18	90%
WAD_1250_-WAD_1500_		19	95%
WAD_1000_-WAD_1250_		12	60%

**Table 5:** Statistical results of the four HIC_15_ values.

Dependent variable	Independent variable	Unstandardized coefficients		Standardized coefficients	*t*	Sig.	Collinearity statistics		*F*	*p*	*R* ^2^
		*B*	Std. error				Tolerance	VIF			
HIC_WAD1125_	*α*	14.634	1.773	0.938	8.255	0.000	0.467	2.142	23.199	0.000	56.0%
	WAD_HFE_	−0.654	0.203	−0.388	−3.221	0.002	0.416	2.406			
	HL	0.275	0.072	0.315	3.848	0.000	0.900	1.112			
	HA	15.238	5.254	0.268	2.900	0.005	0.707	1.414			
	(Constant)	1146.457	230.680		4.970	0.000					
	Regression equation: HIC_WAD1125_ = 14.634 *α* − 0.654 WAD_HFE_ + 0.275 HL + 15.238 HA + 1146.457										

HIC_WAD1375_	WAD_HLE_	1.158	0.132	0.780	8.763	0.000	0.467	2.140	39.557	0.000	73.3%
	HA	25.993	5.771	0.531	4.504	0.000	0.266	3.756			
	HL	0.317	0.056	0.422	5.671	0.000	0.671	1.491			
	*β*	12.742	4.472	0.265	2.849	0.006	0.428	2.337			
	HLER	−0.131	0.049	−0.173	−2.693	0.009	0.898	1.113			
	(Constant)	−915.753	187.012		−4.897	0.000					
	Regression equation: HIC_WAD1375_ = 1.158 WAD_HLE_ + 25.993HA + 0.317HL + 12.742 *β* − 0.131 HLER − 915.753										

HIC_WAD1650_	HR	0.072	0.006	0.929	12.384	0.000	0.651	1.535	49.929	0.000	73.2%
	*γ*	17.030	5.574	0.262	3.055	0.003	0.500	1.999			
	HA	15.240	6.347	0.261	2.401	0.019	0.311	3.217			
	WAD_HLE_	−0.298	0.133	−0.168	−2.241	0.028	0.652	1.535			
	(Constant)	687.282	168.050		4.090	0.000					
	Regression equation: HIC_WAD1650_ = 0.072HR + 17.030*γ* + 15.240HA − 0.298WAD_HLE_ + 687.282 ++										

HIC_WAD2000_	WAD_HRE_	0.909	0.081	0.731	11.225	0.000	0.943	1.061	68.660	0.000	82.4%
	*δ*	23.198	4.476	0.347	5.183	0.000	0.891	1.123			
	HR	0.014	0.006	0.172	2.494	0.016	0.845	1.184			
	(Constant)	−1203.202	168.803		−7.128	0.000					
	Regression equation: HIC_WAD2000_ = 0.909WAD_HRE_ + 23.198*δ* + 0.014HR − 1203.202										

**Table 6:** Correlations between the parameters and the HIC_15_ values and their effect degree.

Styling feature parameters	HIC_WAD1125_	HIC_WAD1375_	HIC_WAD1650_	HIC_WAD2000_
*α*	P^∗∗∗^			
*β*		P		
*γ*			P^∗∗^	
*δ*				P^∗∗^
WAD_HLE_		P^∗∗∗^	N	
WAD_HFE_	N^∗∗^			
WAD_HRE_				P^∗∗∗^
HL	P^∗^	P^∗^		
HA	P	P^∗∗^	P^∗^	
HR			P^∗∗∗^	P^∗^
HLER		N		

P: positive correlation; N: negative correlation; ^∗∗∗^primary factor: the absolute value of the normalization coefficient is the largest in the regression equation; ^∗∗^secondary factor: the absolute value of the normalization coefficient is second in the regression equation; ^∗^tertiary factor: the absolute value of the normalization coefficient is third in the regression equation.

**Table 7:** Result of Pearson's product-moment correlation coefficients.

		HLEH	BL	BCH
WAD_HLE_	Pearson correlation	0.940^∗^	0.608^∗^	0.612^∗^
	Sig. (2-tailed)	0.000	0.000	0.000
	N	78	78	78

WAD_HFE_	Pearson correlation	0.857^∗^	0.511^∗^	0.629^∗^
	Sig. (2-tailed)	0.000	0.000	0.000
	N	78	78	78

^∗^Correlation is significant at the 0.001 level (2-tailed).
